# Rietveld refinement of Y_2_GeO_5_

**DOI:** 10.1107/S1600536809026579

**Published:** 2009-07-15

**Authors:** Eric M. Rivera-Muñoz, Lauro Bucio

**Affiliations:** a Centro de Física Aplicada y Tecnología Avanzada, Universidad Nacional Autónoma de México, AP 1-1010, Queretaro, Qro. 76000, Mexico; b Instituto de Física, Universidad Nacional Autónoma de México, AP 20-364, 01000 México DF, Mexico

## Abstract

Y_2_GeO_5_ (yttrium germanium penta­oxide) was synthesized by solid-state reaction at 1443 K. The arrangement, which has monoclinic symmetry, is isostructural with Dy_2_GeO_5_ and presents two independent sites for the Y atoms. Around these atoms there are distorted six-coordinated YO_6_ octa­hedra and seven-coordinated YO_7_ penta­gonal bipyramids. The YO_7_ polyhedra are linked together, sharing their edges along a surface parallel to *ab*, forming a sheet. Each of these parallel sheets is inter­connected by means of GeO_4_ tetra­hedra, sharing an edge (or vertex) on one side and a vertex (or edge) on the other adjacent side. Parallel sheets of YO_7_ polyhedra are also inter­connected by undulating chains of YO_6_ octa­hedra along the *c* axis. These octa­hedra are joined together, sharing a common edge, to form the chain and share edges with the YO_7_ polyhedra of the sheets.

## Related literature

For the isotypic structure of Dy_2_GeO_5_, see: Brixner *et al.* (1985 ▶). Different synthesis methods have been reported for this compound, including preparation by conventional r.f. magnetron sputtering (Minami *et al.*, 2003 ▶), solid-state reactions at high temperatures (Zhao *et al.*, 2003 ▶), MOCVD and LSMCD (Natori *et al.*, 2004 ▶). For bond-valence parameters, see: Brese & O’Keeffe (1991 ▶), and for the bond-valence model, see: Brown (1981 ▶, 1992 ▶). For oxide phosphors, see: Minami *et al.* (2001 ▶, 2002 ▶, 2004 ▶). Data used to model the second phase present in the reaction product, Y_2_Ge_2_O_7_, were taken from Redhammer *et al.* (2007 ▶). For related literature on technological applications, see: Fei *et al.* (2003 ▶).

## Experimental

### 

#### Crystal data

Y_2_GeO_5_
*M* *_r_* = 330.43
Monoclinic, 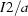

*a* = 10.4706 (2) Å
*b* = 6.8292 (1) Å
*c* = 12.8795 (2) Å
β = 101.750 (3)°
*V* = 901.66 (3) Å^3^
*Z* = 8
Cu *K*α radiation
*T* = 300 K
Specimen shape: flat sheet
20 × 20 × 0.2 mm
Specimen prepared at 1443 K
Particle morphology: spherical, white

#### Data collection

Bruker Advance D8 diffractometer
Specimen mounting: packed powder sample container
Specimen mounted in reflection mode
Scan method: step
2θ_min_ = 8.0, 2θ_max_ = 80.0°
Increment in 2θ = 0.02°

#### Refinement

*R*_p_ = 0.053
*R*_wp_ = 0.069
*R*_exp_ = 0.024
*S* = 2.90
Wavelength of incident radiation: 1.540560 Å
Profile function: pseudo-Voigt modified by Thompson *et al.* (1987 ▶)
582 reflections
105 parameters

### 

Data collection: *DIFFRAC/AT* (Siemens, 1993 ▶); cell refinement: *DICVOL91* (Boultif & Louëer 1991 ▶); data reduction: *FULLPROF* (Rodríguez-Carvajal, 2006 ▶); method used to solve structure: coordinates taken from an isotypic compound (Brixner *et al.*, 1985 ▶); program(s) used to refine structure: *FULLPROF*; molecular graphics: *ATOMS* (Dowty, 2000 ▶); software used to prepare material for publication: *FULLPROF*.

## Supplementary Material

Crystal structure: contains datablocks global, I. DOI: 10.1107/S1600536809026579/br2110sup1.cif

Rietveld powder data: contains datablocks I. DOI: 10.1107/S1600536809026579/br2110Isup2.rtv

Additional supplementary materials:  crystallographic information; 3D view; checkCIF report

## Figures and Tables

**Fig. 1.**
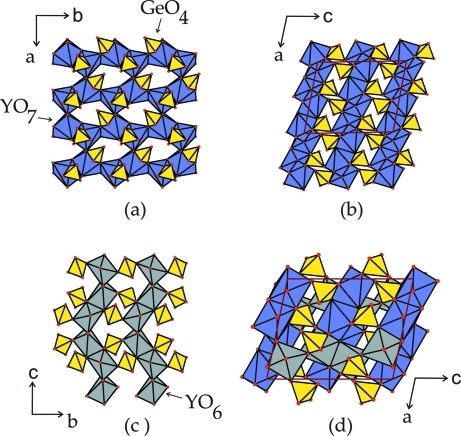
(a) View of a YO7 layer in the Y2GeO5 structure (ab projection). YO7 polyhedra are represented in medium slate blue while GeO4 tetrahedra are represented in yellow. (b) View of the Y2GeO5 structure (ac projection). The layers formed by YO7 polyhedra are linked together by GeO4 tetrahedra. (c) Chains of YO6 octahedra (in gray) undulating along the c axis sharing common edges and linked with other chains by mean of GeO4 tetrahedra. (d) Unit cell for Y2GeO5 structure.

**Fig. 2.**
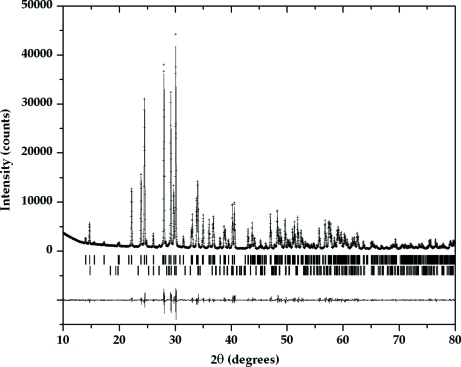
Rietveld refinement for X-ray diffraction data. Observed (crosses), calculated (solid line) and difference (bottom trace) plots are represented; vertical marks correspond to the allowed Bragg reflections for Y2GeO5 (top) and Y2Ge2O7 (bottom) as secondary phase.

## supplementary crystallographic information

### Comment

Field emission display (FED) constitutes the next generation of information display devices. Its advantages include portable size with low power consumption, broad viewing angle, and wide operating-temperature range among others (Zhao *et al.*, 2003). New multicomponent oxide phosphor, Mn-activated Y_2_O_3_—GeO_2_, is promising as the thin-film emitting layer for thin-film electroluminescent (TFEL) devices (Minami *et al.*, 2001). The oxide phosphor for use in those electroluminescent devices is formed from yttrium oxide and a transition metal as an activator, or from Y—Ge—O oxide and one metallic element to form *M*:Y_2_GeO_5_ where *M* is a metal (Minami *et al.*, 2002; Minami *et al.*, 2004). Other reported use for Y_2_GeO_5_ consists in piezoelectric ceramics in the form of films which include a complex oxide material having an oxygen octahedral structure and a paraelectric material having a catalytic effect for the complex oxide material in a mixed state. Paraelectric material could be a layered compound having an oxygen tetrahedral structure which includes one compound with the form MSiO*_x_* (*M*=metal) and Y_2_GeO_5_ (Natori *et al.*, 2004). Fig. 1a show a fragment of the crystal structure of Y_2_GeO_5_ along the *ab* plane in which YO_7_ polyhedra share common edges forming a mesh. These YO_7_ polyhedra are represented as medium slate blue. Over the mesh, there are isolated GeO_4_ tetrahedra, which are represented in yellow in Fig. 1a. Each one of these parallel sheets are interconnected by means of GeO_4_ tetrahedra, sharing an edge (or vertix) in one side and a vertix (or edge) in the other adjacent side respectively as can be seen in Fig. 1b. Undulating chains of YO_6_ octahedra along the *c* axis are represented in gray in Fig. 1c in which the YO_7_ polyhedra were not represented in order lo clarify this feature of the arrangement. The chains of YO_6_ octahedra also interconnect the parallel sheets of YO_7_ polyhedra, as can be see in the unit cell of Y_2_GeO_5_ represented in Fig. 1d. Bond valence calculations were made using the recommended bond-valence parameters for oxides published by Brese & O'Keeffe (1991). Bond valence sum (BVS) around six-coordinated Y1, seven-coordinated Y2, and Ge give the values of 3.03, 2.76 and 4.08 respectively, being the first and the last closer to the values of +3 and +4 expected for the yttrium and germanium atoms respectively. The second value of 2.76 was first interpreted as stretched bonds around Y2 exist, but this suggestion was withdrawn because there is no compressed cation in the unit cell capable to balance the supposed stretched bonds around Y2, as it is established in the Brown's bond valence model (Brown, 1981) for evaluating the existence of stresses in the crystal.  In fact, calculating the so called *Global Instability Index*, which is obtained as the root mean square of the bond-valence sum deviation for all the N atoms present in the asymmetric unit (Brown, 1992) a value of 0.06 was obtained suggesting no strain. This is a remarkably low value for a Rietveld refinement (for a well refined and unstrained structure this is less than 0.1).  Then, the low value of the bond valence sum around Y2 is well within normal limits for a Rietveld refinment where larger deviations are typically found.

### Experimental

The reactive mixture was prepared from Y_2_O_3 _(Aldrich.99.99%) and GeO_2_ (CERAC 99.999%) according to the stoichiometric proportions desired. The mixture was first powdered using an agate mortar; and then was heated in air in a tube furnace at 1373 K for 5 days with intermediate regrindings. A second thermal treatment at 1443 K for two days was applied. The characterization of the bulk material by conventional X-ray powder diffraction data indicated the presence of a well crystallized phase showing reflections that match with the isostructural phase DyGeO_5_ (PDF 01–078–0478). Very small amount of a secondary phase Y_2_Ge_2_O_7_ (PDF 38–288) was identified.

### Refinement

The starting structural parameters for perform a Rietveld refinement of the Y_2_GeO_5_ phase were taken from the isostructural data reported for Dy_2_GeO_5_ (ICSD 61373) by Brixner *et al.* (1985). For modeling the second phase Y_2_Ge_2_O_7_ (ICSD 240989), the data were those reported by Redhammer *et al.* (2007). The following parameters were refined: zero point and scale factors, cell parameters, half-width profile parameters, overall temperature factors, atomic coordinates, and asymmetries. For the Y_2_Ge_2_O_7 _phase the atomic coordinates were fixed to their starting values. The final Rietveld refinement of conventional diffraction pattern is shown in Fig. 2.

### Crystal data

**Table:**

Y_2_GeO_5_	*Z* = 8
*M**_r_* = 330.43	*F*(000) = 1200
Monoclinic, *I*2/*a*	*D*_x_ = 4.868 Mg m^−^^3^
Hall symbol: -I 2ya	Cu *K*α radiation, λ = 1.540560 Å
*a* = 10.4706 (2) Å	*T* = 300 K
*b* = 6.8292 (1) Å	Particle morphology: spherical
*c* = 12.8795 (2) Å	white
β = 101.750 (3)°	flat sheet, 20 × 20 mm
*V* = 901.66 (3) Å^3^	Specimen preparation: Prepared at 1443 K

### Data collection

**Table:**

Bruker Advance D8 diffractometer	Data collection mode: reflection
Radiation source: sealed X-ray tube, Cu Kα	Scan method: step
graphite	2θ_min_ = 7.98°, 2θ_max_ = 80.00°, 2θ_step_ = 0.02°
Specimen mounting: packed powder sample container	

### Refinement

**Table:**

Least-squares matrix: full with fixed elements per cycle	Profile function: pseudo-Voigt modified by Thompson *et al.* (1987)
*R*_p_ = 0.053	105 parameters
*R*_wp_ = 0.069	Weighting scheme based on measured s.u.'s
*R*_exp_ = 0.024	(Δ/σ)_max_ = 0.02
χ^2^ = 8.410	Background function: The background was refined first by mean of a linear interpolation between 55 background points with adjustable heights. At the end of the refinement, the values for all of these heights of the background were fixed.
3704 data points	

### Fractional atomic coordinates and isotropic or equivalent isotropic displacement parameters (Å^2^)

**Table:**

	*x*	*y*	*z*	*U*_iso_*/*U*_eq_	
Y1	0.3011 (2)	0.6277 (2)	0.6380 (1)	0.0096 (7)	
Y2	0.0708 (2)	0.2567 (3)	0.5355 (1)	0.0090 (7)	
Ge1	0.6236 (2)	0.5933 (3)	0.8155 (2)	0.0121 (9)	
O1	0.1210 (9)	0.604 (1)	0.5178 (8)	0.009 (2)	
O2	0.2950 (9)	0.298 (1)	0.6172 (7)	0.009 (2)	
O3	0.5212 (9)	0.654 (1)	0.6971 (8)	0.009 (2)	
O4	0.551 (1)	−0.006 (1)	0.4155 (8)	0.009 (2)	
O5	0.2412 (8)	0.572 (1)	0.7926 (8)	0.009 (2)	

### Geometric parameters (Å, °)

**Table:**

Y1—O1	2.189 (8)	Y2—O2^i^	2.655 (10)
Y1—O1^i^	2.321 (10)	Y2—O3^iv^	2.327 (10)
Y1—O2	2.270 (8)	Y2—O4^i^	2.358 (9)
Y1—O3	2.283 (8)	Y2—O4^v^	2.287 (9)
Y1—O5	2.238 (10)	Ge1—O2^vi^	1.767 (8)
Y1—O5^ii^	2.316 (8)	Ge1—O3	1.727 (8)
Y2—O1	2.447 (8)	Ge1—O4^vii^	1.732 (10)
Y2—O1^iii^	2.203 (8)	Ge1—O5^viii^	1.739 (9)
Y2—O2	2.386 (8)		
			
Y1—O1—Y1^ix^	53.6 (2)	Y1—O2—Y2^xiii^	118.8 (3)
Y1—O1—Y2^x^	128.7 (3)	Y2^xii^—O2—Y2^xiii^	61.19 (6)
Y1^ix^—O1—Y2^x^	78.64 (6)	Y1—O3—Y2^xiv^	110.7 (3)
Y1^ix^—O1—Y2^xi^	90.44 (7)	Y2^xv^—O4—Y2^xvi^	124.20 (7)
Y2^x^—O1—Y2^xi^	157.05 (6)	Y1—O5—Y1^xvii^	89.8 (2)
Y1—O2—Y2^xii^	97.4 (2)		

Symmetry codes: (i) −*x*+1/2, *y*, −*z*+1; (ii) −*x*+1/2, −*y*+3/2, −*z*+3/2; (iii) −*x*, −*y*+1, −*z*+1; (iv) *x*−1/2, −*y*+1, *z*; (v) *x*−1/2, −*y*, *z*; (vi) −*x*+1, *y*+1/2, −*z*+3/2; (vii) *x*, −*y*+1/2, *z*+1/2; (viii) *x*+1/2, −*y*+1, *z*; (ix) −*x*+3/2, *y*, −*z*+1; (x) −*x*, −*y*, −*z*; (xi) *x*+1/2, −*y*+1, *z*+1; (xii) −*x*+1/2, *y*, −*z*; (xiii) −*x*+1, −*y*, −*z*+1; (xiv) −*x*+3/2, *y*+1, −*z*; (xv) *x*+1, *y*, *z*+1; (xvi) −*x*+3/2, *y*, −*z*; (xvii) −*x*+3/2, *y*+2, −*z*+2.
